# Can Delta Radiomics Improve the Prediction of Best Overall Response, Progression-Free Survival, and Overall Survival of Melanoma Patients Treated with Immune Checkpoint Inhibitors?

**DOI:** 10.3390/cancers16152669

**Published:** 2024-07-26

**Authors:** Felix Peisen, Annika Gerken, Alessa Hering, Isabel Dahm, Konstantin Nikolaou, Sergios Gatidis, Thomas K. Eigentler, Teresa Amaral, Jan H. Moltz, Ahmed E. Othman

**Affiliations:** 1 Department of Diagnostic and Interventional Radiology, Eberhard Karls University, Tuebingen University Hospital, Hoppe-Seyler-Straße 3, 72076 Tuebingen, Germany; isabel.dahm@med.uni-tuebingen.de (I.D.); konstantin.nikolaou@med.uni-tuebingen.de (K.N.); sergios.gatidis@med.uni-tuebingen.de (S.G.); 2 Fraunhofer Institute for Digital Medicine MEVIS, Max-von-Laue-Straße 2, 28359 Bremen, Germany; annika.gerken@mevis.fraunhofer.de (A.G.); alessa.hering@mevis.fraunhofer.de (A.H.); jan.moltz@mevis.fraunhofer.de (J.H.M.); 3 Diagnostic Image Analysis Group, Radboudumc, Geert Grooteplein Zuid 10, 6525 GA Nijmegen, The Netherlands; 4 Cluster of Excellence iFIT (EXC 2180) “Image-Guided and Functionally Instructed Tumor Therapies”, Faculty of Medicine, Eberhard Karls University, 72076 Tuebingen, Germany; 5 Max Planck Institute for Intelligent Systems, Max-Planck-Ring 4, 72076 Tuebingen, Germany; 6 Center of Dermato-Oncology, Department of Dermatology, Eberhard Karls University, Tuebingen University Hospital, Liebermeisterstraße 25, 72076 Tuebingen, Germany; thomas.eigentler@charite.de (T.K.E.); teresa.amaral@med.uni-tuebingen.de (T.A.); 7 Department of Dermatology, Venereology and Allergology, Charité—Universitätsmedizin Berlin, Freie Universität Berlin and Humboldt-Universität zu Berlin, Luisenstraße 2, 10117 Berlin, Germany; 8 Institute of Neuroradiology, Johannes Gutenberg University Hospital Mainz, Langenbeckstraße 1, 55131 Mainz, Germany; ahmed.othman@unimedizin-mainz.de

**Keywords:** immunotherapy, melanoma, total tumour burden, volumetric segmentation, delta radiomics, prediction, response, survival

## Abstract

**Simple Summary:**

The incidence of metastatic melanoma is rising, making it imperative to identify patients who do not benefit from immunotherapy. This study aimed to develop a radiomic biomarker, using segmentations from 146 baseline and 146 first follow-up CT scans, to predict best overall response, progression-free survival, and overall survival across various immunotherapies. We volumetrically segmented the total tumour load, excluding cerebral metastases. This study also examined whether reducing the number of segmented metastases per patient affects predictive accuracy. The findings suggest that delta radiomics could enhance the prediction of best overall response, progression-free survival, and overall survival in metastatic melanoma patients undergoing first-line immunotherapy. Although volumetric whole tumour load segmentation is complex, it may provide predictive benefits.

**Abstract:**

Background: The prevalence of metastatic melanoma is increasing, necessitating the identification of patients who do not benefit from immunotherapy. This study aimed to develop a radiomic biomarker based on the segmentation of all metastases at baseline and the first follow-up CT for the endpoints best overall response (BOR), progression-free survival (PFS), and overall survival (OS), encompassing various immunotherapies. Additionally, this study investigated whether reducing the number of segmented metastases per patient affects predictive capacity. Methods: The total tumour load, excluding cerebral metastases, from 146 baseline and 146 first follow-up CTs of melanoma patients treated with first-line immunotherapy was volumetrically segmented. Twenty-one random forest models were trained and compared for the endpoints BOR; PFS at 6, 9, and 12 months; and OS at 6, 9, and 12 months, using as input either only clinical parameters, whole-tumour-load delta radiomics plus clinical parameters, or delta radiomics from the largest ten metastases plus clinical parameters. Results: The whole-tumour-load delta radiomics model performed best for BOR (AUC 0.81); PFS at 6, 9, and 12 months (AUC 0.82, 0.80, and 0.77); and OS at 6 months (AUC 0.74). The model using delta radiomics from the largest ten metastases performed best for OS at 9 and 12 months (AUC 0.71 and 0.75). Although the radiomic models were numerically superior to the clinical model, statistical significance was not reached. Conclusions: The findings indicate that delta radiomics may offer additional value for predicting BOR, PFS, and OS in metastatic melanoma patients undergoing first-line immunotherapy. Despite its complexity, volumetric whole-tumour-load segmentation could be advantageous.

## 1. Introduction

Melanoma, particularly in its metastatic stage IV form, is increasingly diagnosed [1,2]. Since the advent of immune checkpoint inhibitors like PD-1 (programmed death-1) inhibitors (nivolumab and pembrolizumab), and CTLA-4 (cytotoxic T-lymphocyte-associated protein 4) inhibitors (ipilimumab), prognoses have improved significantly [3,4,5,6,7]. However, treatment resistance and severe side effects such as autoimmune pancreatitis and pneumonitis pose significant challenges [8,9,10]. Therefore, there is a critical need to identify patients who do not benefit from these therapies to guide them towards more effective alternatives. Radiomics, which extracts large datasets from imaging studies and translates them into biomarkers, has shown promise in this regard [11,12,13]. Initial radiomic applications include pre-treatment imaging with CT (computed tomography) or 18F-FDG-PET/CT (flourine-18 fluorodeoxyglucose positron emission tomography/computed tomography). Some studies have demonstrated the predictive power of baseline CT radiomics for survival in melanoma patients [14,15,16,17] although their results have been inconsistent [18].

Delta radiomics, which evaluates changes in imaging features over time, offers an advanced approach. This method combines baseline and follow-up imaging data to generate predictive biomarkers [19,20,21]. Previous studies have shown the potential of delta radiomics in predicting outcomes such as overall survival and progression-free survival in small cohorts of melanoma patients. Guerrisi et al. and Wang et al. performed pilot studies in small groups of patients with malignant melanoma and reported that CT delta texture analysis predicted overall survival and progression-free survival as well as early response to immunotherapy and pseudo progression [21,22]. Dercle et al. studied a larger cohort undergoing immunotherapy with pembrolizumab. They followed a whole-tumour-load segmentation approach on baseline and first follow-up CTs and published a radiomic signature for predicting overall survival. However, the reported time required for manual lesion segmentation of approximately one minute per lesion per scan, and the restriction to pembrolizumab monotherapy limits the clinical application of the signature [23].

This study aimed to evaluate whether a more generalizable radiomic biomarker could be developed for the endpoints BOR, PFS, and OS, using a larger and more diverse patient sample. Additionally, it explored whether reducing the number of segmented metastases affects predictive performance.

## 2. Materials and Methods

### 2.1. Patients

**Figure 1:**
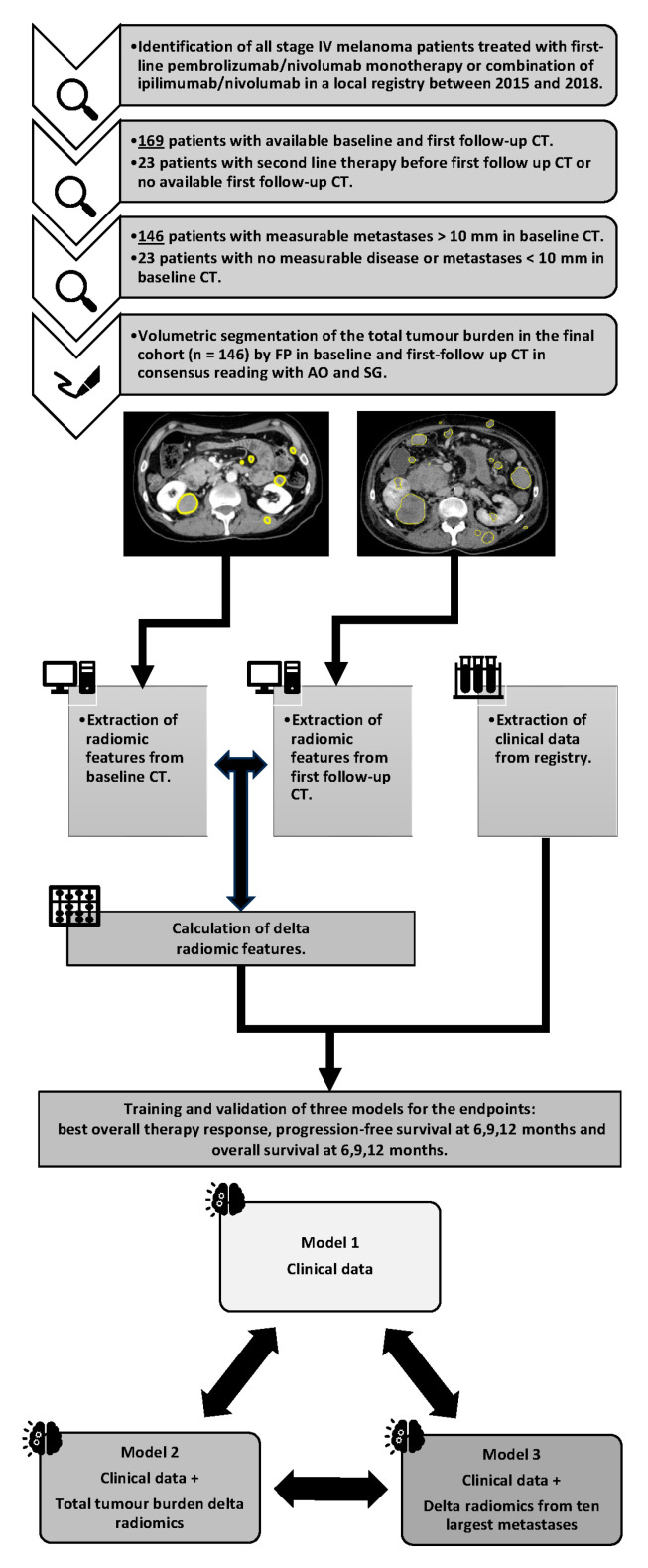
Workflow diagram.

This study included patients with stage IV malignant melanoma (AJCC 8th edition [24]) treated between 2015 and 2018, as recorded in the local dermatology melanoma registry. Patients treated with first-line immunotherapy (PD-1 checkpoint inhibitor monotherapy or combination of a PD-1 checkpoint inhibitor and a CTLA-4 checkpoint inhibitor), who had available contrast-enhanced baseline and first follow-up CT imaging and measurable disease at baseline were included. The study protocol received institutional review board approval (protocol code 092/2019BO2, 21 February 2019), and informed consent was waived due to the retrospective design. A workflow diagram is shown in Figure 1.

### 2.2. Imaging

Baseline and first follow-up CTs were retrieved from the local picture archiving and communication system (PACS), anonymised, and uploaded into custom software (SATORI, Fraunhofer MEVIS, Bremen, Germany) for volumetric segmentation of all measurable metastases. A radiologist (F.P.) with six years of oncologic imaging experience conducted manual segmentation for baseline CTs in consensus reading with A.E.O. and S.G. (both specialists in oncologic imaging), while follow-up CT segmentations were precomputed by an algorithm trained on baseline segmentations [25] and reviewed by the same radiologists. A detailed distribution of the CT scanners and imaging parameters can be found in Table S1 in Supplementary Materials. Examples of different timepoint responses can be depicted from Figure S1. Radiomic feature extraction was performed using the Pyradiomics Python package (v3.1.0) [11], and delta features were computed at a patient level. A detailed description of the radiomic feature extraction and aggregation is provided in Supplementary Materials.

### 2.3. Model Development

Random forest models were trained for seven different clinical endpoints (best overall response to therapy according to Response Evaluation Criteria In Solid Tumors (RECIST) 1.1 criteria [26] (binarised: complete or partial response = response; stable or progressive disease = no response); progression-free survival after six, nine, and twelve months; and overall survival after six, nine, and twelve months. Model development and validation were conducted using Python, version 3.6.13 (Python Software Foundation, Beaverton, OR, USA).

### 2.4. Validation

Considering the conclusions of Kocak et al. [27], model performance was validated against a clinical parameter-only model that used the following features as input: age, gender, type of immunotherapy, localization of primary tumour, histological subtype of primary tumour, BRAF (v-Raf murine sarcoma viral oncogene homolog B1) V600E mutation status, baseline lactate dehydrogenase level, follow-up lactate dehydrogenase level, baseline S100 level, follow-up S100 level, number of metastatic organs in baseline CT, and presence of cerebral metastases or hepatic metastases and presence of new metastases in first follow-up CT. Tests were also conducted against a model using only the ten largest metastases per patient for the extraction of radiomic features, simulating a more realistic segmentation approach from a clinical perspective. Performance was estimated using ten-time-repeated five-fold cross-validation. For a detailed description of the radiomics feature extraction and aggregation, the machine learning model, and the model evaluation, see Supplementary Materials S2.1–S2.3.

### 2.5. Statistical Analysis

Analyses were conducted using Excel, version 2019 (Microsoft Corporation, Redmond, DC, USA), SPSS Statistics 29 (IBM, Armonk, NY, USA), and R, version 3.6.2 (R Program for Statistical Computing, Vienna, Austria). The area under the curve (AUC) of the receiver-operating-characteristic (ROC) curve was used as a classification performance metric. Statistically significant superior performance of the extended model was achieved if the 95% confidence intervals (CI) of the mean AUC of the baseline and extended models did not overlap. Significant predictive capacity of a model following the outcome distribution was achieved if the lower bound of the CI was higher than 0.5.

## 3. Results

### 3.1. Patients’ Characteristics

The final cohort consisted of 146 patients, predominantly male (63%), with a median age of 66 years. The most common histological subtype was nodular melanoma (28%). Most patients received either nivolumab and ipilimumab combination therapy (45%) or pembrolizumab monotherapy (42%). A detailed description of the patients’ characteristics is shown in Table 1.

### 3.2. Random Forest Models for Binarised Best Overall Therapy Response

**Figure 2:**
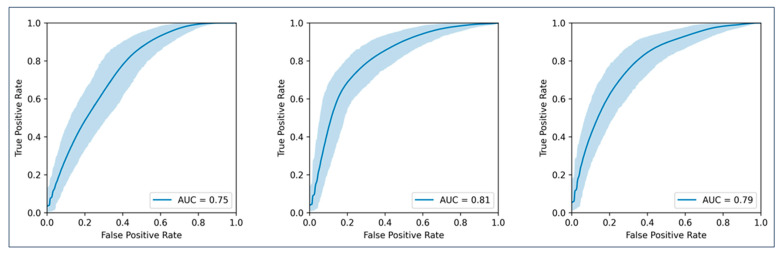
AUCs for the prediction of binarised best overall response. **Left graph** represents the model using only clinical parameters; **middle graph** represents the model using clinical parameters plus radiomic features from all metastases per patient; **right graph** represents the model using clinical parameters plus radiomic features from the largest ten metastases per patient.

Three random forest models were cross-validated for binarised BOR (see Figure 2). The model using clinical data and whole-tumour burden radiomics achieved the highest AUC (0.81), followed by the model using clinical data and radiomics from the ten largest metastases (AUC 0.79). The clinical data-only model achieved an AUC of 0.75. Detailed values are shown in Table 2. As the confidence intervals of the clinical and radiomics models overlapped, statistical significance was not reached according to our definition.

### 3.3. Random Forest Models for Progression-Free Survival

**Figure 3:**
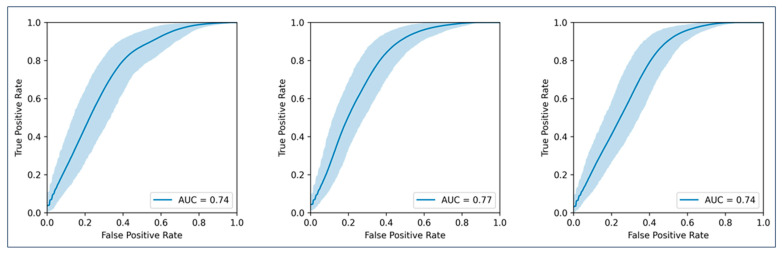
AUCs for the prediction of progression-free survival at twelve months. (**Left graph**) represents the model using only clinical parameters; (**middle graph**) represents the model using clinical parameters plus radiomic features from all metastases per patient; (**right graph**) represents the model using clinical parameters plus radiomic features from the largest ten metastases per patient.

Nine models were cross-validated for PFS at 6, 9, and 12 months (Figure 3 shows the endpoint PFS at 12 months). The best performance was achieved with models combining clinical data and radiomics from the whole-tumour burden (AUCs 0.82, 0.80, and 0.77 for 6, 9, and 12 months, respectively). Detailed values are shown in Table 2. As the confidence intervals of the clinical and radiomics models overlapped, statistical significance was not reached according to our definition.

### 3.4. Random Forest Models for Overall Survival

**Figure 4:**
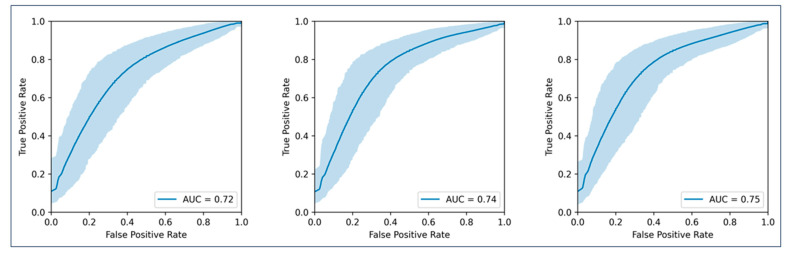
AUC curves for the prediction of overall survival for twelve months. (**Left graph**) represents the model using only clinical parameters; (**middle graph**) represents the model using clinical parameters plus radiomic features from all metastases per patient; (**right graph**) represents the model using clinical parameters plus radiomic features from the largest ten metastases per patient.

Nine models were cross-validated for OS at 6, 9, and 12 months (Figure 4 shows the endpoint OS at 12 months). The model for OS at 6 months performed best with whole-tumour-burden radiomics (AUC 0.74). Models for OS at 9 and 12 months performed best with radiomics from the ten largest metastases (AUC 0.71 and 0.75, respectively). Detailed values are shown in Table 2. As the confidence intervals of the clinical and radiomics models overlapped, statistical significance was not reached according to our definition.

## 4. Discussion

Radiomics offers promising potential for identifying melanoma patients who may not benefit from immunotherapy. Several studies have reported that relevant features can be identified from baseline CT imaging. The delta approach may further enhance predictive capabilities of radiomics, as demonstrated by data published by Dercle et al. [23]. However, the proposed algorithm lacks clinical transferability as it is limited to pembrolizumab therapy and requires segmentation of the entire tumour burden on baseline and follow-up imaging. Therefore, our aim was to develop a model applicable to a wider range of immunotherapies and to investigate whether the quantity of lesions required for segmentation could be reduced to a more manageable number.

This study demonstrated that delta radiomics of the whole-tumour volume improved model performance for most endpoints (BOR; PFS 6, 9, 12 months; and OS 6 months). The AUCs were all numerically superior compared to a model using only clinical features. Compared to the results published by Dercle et al. [23], our AUC values for the prediction of OS were lower (0.74–0.70 compared to 0.92). The reason for this discrepancy is most likely the composition of the investigated samples studied. Dercle et al. restricted their cohort to melanoma patients treated with a single agent programmed cell death 1 blocker (pembrolizumab). In contrast, we aimed for a more generalizable approach and included patients with pembrolizumab or nivolumab monotherapy or combined nivolumab/ipilimumab therapy to allow for improved clinical applicability. The positive effect of delta radiomics was still present in our approach, but it should be noted that the confidence intervals for all endpoints overlapped. Therefore, according to our definition, there was a numerical improvement, but not a statistically significant difference.

Manual volumetric segmentation of the whole-tumour burden is very time-consuming. At approximately one minute per lesion per scan, a patient with multifocal metastatic melanoma and only thirty metastases will require an hour to segment a baseline and follow-up scan. This approach is highly problematic in terms of clinical application. Reducing the number of segmented lesions to the ten largest metastases still provided valuable information but with slightly lower predictive performance, except for OS 9 and 12 months. This shows that segmentation of the entire tumour burden, although time-consuming, may provide more information. Advances in AI-enhanced segmentation may mitigate these challenges, making volumetric segmentation more feasible in clinical settings. Several techniques for automated or semi-automated lesion segmentation have been reported [25,28,29,30]. Hering et al. published two studies that proved the feasibility of automated tracking and volumetric segmentation using the example of soft tissue and lymph node metastases. They could show that their proposed pipeline with a so called initial “one-click” segmentation at baseline imaging and subsequent automated segmentation in follow-up imaging is non-inferior to manual segmentation, significantly saves time, and reduces inter-reader variability [25,30]. The pipeline is based on the nnU-Net by Isensee et al., a deep learning framework, that showed impressive results at segmentation tasks, using a self configurating architecture [29]. Moawad et al. nicely discussed the limitations of the U-Net architecture for 3D medical imaging segmentation and potential solutions, such as variants that accept 3D imaging as input (V-Net, 3D U-Net, hybrid Dense U-Net) or different architectures such as fully convolutional networks (FCN) [28]. Although not all these methods have become established in daily routine image reading, segmentation of the entire tumour burden in a manageable amount of time is theoretically no longer unrealistic and might enable radiologists to access additional information, such as radiomics or volumetric RECIST [31,32].

Our study has strengths and limitations. We used a registry with prospective data documentation to identify the sample studied. The cohort contained a large number of patients treated according to current guidelines at a tertiary referral centre. Volumetric segmentation was carried out by an experienced radiologist in consensus reading with two experts in oncological imaging. Prospective validation on an external dataset and a second segmentation by a second reader are lacking. A larger sample size would have been beneficial, and we are hoping to expand our dataset in the future.

## 5. Conclusions

Overall, delta radiomics shows potential for improving the prediction of BOR, PFS, and OS in metastatic melanoma patients receiving first-line immunotherapy. Despite its complexity, a volumetric segmentation of the whole-tumour burden could be favourable. Further research with larger cohorts and prospective validation is needed to confirm these findings and enhance clinical applicability.

## Figures and Tables

**Table 1:** Patient’s characteristics.

Clinical Data		
Age (years) [median, (IQR)]		66 (22%)
Gender (male) [*n*, %]		92 (63%)
Localization of primary tumour [*n*, %]	head/neck	34 (23%)
	torso	35 (24%)
	upper extremity	21 (14%)
	lower extremity	38 (26%)
	other	7 (5%)
	n/a	11 (8%)
Histological subtype [*n*, %]	SSM	35 (24%)
	NM	41 (28%)
	LMM	8 (5%)
	ALM	22 (14%)
	mucosal	7 (5%)
	occult	9 (6%)
	n/a	27 (18%)
BRAF V600E mutation status [*n*, %]	BRAF wildtype	101 (69%)
	BRAF mutation	41 (28%)
	n/a	4 (3%)
Baseline LDH [*n*, %]	normal (<250 U/L)	31 (21%)
	elevated (≥250 U/L)	100 (69%)
	n/a	15 (10%)
FU1 LDH [*n*, %]	normal (<250 U/L)	86 (59%)
	elevated (≥250 U/L)	57 (39%)
	n/a	3 (2%)
Baseline S100B [*n*, %]	normal (<0.1 µg/L)	77 (53%)
	elevated (≥0.1 µg/L)	59 (40%)
	n/a	10 (7%)
FU1 S100B [*n*, %]	normal (<0.1 µg/L)	80 (55%)
	elevated (≥0.1 µg/L)	61 (42%)
	n/a	5 (3%)
Number of metastatic organs [*n*, %]	1–3	132 (90%)
	> 3	14 (1%)
Presence of cerebral metastases [*n*, %]		23 (16%)
Presence of hepatic metastases [*n*, %]		39 (27%)
Therapy [*n*, %]	pembrolizumab	61 (42%)
	nivolumab	19 (13%)
	nivolumab + ipilimumab	66 (45%)
Baseline CT lesion counts [*n*]	all	3188
	lung	1411
	liver	584
	soft tissue/skin	416
	lymph nodes	478
	skeletal	77
	spleen	74
	other	148
FU1 CT lesion counts [*n*]	all	4836
	lung	2104
	liver	1083
	soft tissue/skin	707
	lymph nodes	588
	skeletal	71
	spleen	92
	other	191
Patient outcome		
Best overall response (RECIST 1.1) [*n*, %]	CR	26 (18%)
	PR	46 (31%)
	SD	22 (15%)
	PD	48 (33%)
	n/a	4 (3%)
Progression-free survival for 6 months [*n*, %]	yes	68 (44%)
	no	66 (48%)
	n/a	12 (8%)
Progression-free survival for 9 months [*n*, %]	yes	54 (37%)
	no	73 (50%)
	n/a	19 (13%)
Progression-free survival for 12 months [*n*, %]	yes	41 (28%)
	no	77 (53%)
	n/a	28 (19%)
Overall survival after 6 months [*n*, %]	yes	110 (75%)
	no	19 (13%)
	n/a	17 (12%)
Overall survival after 9 months [*n*, %]	yes	90 (62%)
	no	27 (18%)
	n/a	29 (20%)
Overall survival after 12 months [*n*, %]	yes	70 (48%)
	no	30 (21%)
	n/a	46 (31%)

Abbreviations: ALM, acral lentiginous melanoma; CR, complete response;; FU1, first follow-up, IQR, interquartile range; LDH, lactate dehydrogenase; LMM, lentigo maligna melanoma; n/a, not available; NM, nodular melanoma;; PD, progressive disease;, PR, partial response; RECIST, Response Evaluation Criteria In Solid Tumors; SD, stable disease; SSM, superficial spreading melanoma.

**Table 2:** Number of cases for the different endpoints with class distributions and mean AUC from a 10 × 5-fold CV and 95% confidence interval computed by bootstrapping the 10 × 5-fold CV. Class 0 for BOR = PD/SD; class 1 for BOR = PR/CR; class 0 for PFS/OS = no; class 1 for PFS/OS = yes.

Binary Endpoint	Cases *n* (Class 0 + 1)	Model with Clinical Features Only. AUC (95%CI)	Model with Clinical Features + Whole- Tumour-Load Radiomic Features. AUC (95%CI)	Model with Clinical Features + Radiomic Features from Largest Ten Lesions. AUC (95%CI)
Best overall response	142 (70 + 72)	0.750 (0.672, 0.822)	0.811 (0.745, 0.876)	0.794 (0.726, 0.862)
PFS 6 months	134 (66 + 68)	0.797 (0.726, 0.859)	0.824 (0.756, 0.882)	0.814 (0.747, 0.874)
PFS 9 months	127 (73 + 54)	0.764 (0.684, 0.832)	0.797 (0.730, 0.855)	0.774 (0.702, 0.841)
PFS 12 months	118 (77 + 41)	0.742 (0.658, 0.816)	0.769 (0.698, 0.839)	0.741 (0.667, 0.815)
OS 6 months	129 (19 + 110)	0.721 (0.588, 0.848)	0.742 (0.598, 0.870)	0.718 (0.583, 0.852)
OS 9 months	117 (27 + 90)	0.684 (0.568, 0.788)	0.704 (0.594, 0.808)	0.708 (0.590, 0.811)
OS 12 months	101 (31 + 70)	0.724 (0.617, 0.822)	0.744 (0.642, 0.836)	0.746 (0.641, 0.838)

Abbreviations: AUC, area under the curve; BOR, best overall response; CI, confidence interval; CR, complete response; CV, cross-validation; *n*, number; OS, overall survival; PD, progressive disease; PFS, progression-free survival; PR, partial response; SD, stable disease.

## Data Availability

The complete dataset cannot be made freely available to the public, due to privacy regulations. A minimal dataset, codes, and materials used in this study may be made available for purposes of reproducing or extending the analysis, pending material transfer agreements, after a reasonable and well justified request to Felix Peisen.

## Supplementary Materials

The following supporting information can be downloaded at https://www.mdpi.com/article/10.3390/cancers16152669/s1. Scan parameters and CT vendor details; Detailed description of the radiomic feature extraction and aggregation, the machine learning model, and model evaluation; Table S1: CT scanners and vendors. Figure S1: Examples of different timepoint responses: A, C, E show baseline CT imaging of three different patients. B, D, F show CT imaging of the first follow-up with timepoint responses “partial response”, “stable disease” and “progressive disease”, respectively.

## Abbreviations

ALM	acral lentiginous melanoma
AUC	area under the curve
BRAF	v-Raf murine sarcoma viral oncogene homolog B1
CI	confidence interval
CR	complete response
CT	computed tomography
CTLA-4	cytotoxic T-lymphocyte-associated protein 4
CV	cross-validation
IQR	interquartile range
LDH	lactate dehydrogenase
LMM	lentigo maligna melanoma
n	number
NM	nodular melanoma
OS	overall survival
PACS	picture archiving and communication system
PD	progressive disease
PD-1	programmed death 1
PET	positron emission tomography
PFS	progression-free survival
PR	partial response
RECIST	Response Evaluation Criteria In Solid Tumors
ROC	receiver operating characteristic
SD	stable disease
SSM	superficial spreading melanoma
