# Correction: Peisen et al. Can Delta Radiomics Improve the Prediction of Best Overall Response, Progression-Free Survival, and Overall Survival of Melanoma Patients Treated with Immune Checkpoint Inhibitors? *Cancers* 2024, *16*, 2669

**DOI:** 10.3390/cancers17010001

**Published:** 2024-12-24

**Authors:** Felix Peisen, Annika Gerken, Alessa Hering, Isabel Dahm, Konstantin Nikolaou, Sergios Gatidis, Thomas K. Eigentler, Teresa Amaral, Jan H. Moltz, Ahmed E. Othman

**Affiliations:** 1 Department of Diagnostic and Interventional Radiology, Eberhard Karls University, Tuebingen University Hospital, Hoppe-Seyler-Straße 3, 72076 Tuebingen, Germany; isabel.dahm@med.uni-tuebingen.de (I.D.); konstantin.nikolaou@med.uni-tuebingen.de (K.N.); sergios.gatidis@med.uni-tuebingen.de (S.G.); 2 Fraunhofer Institute for Digital Medicine MEVIS, Max-von-Laue-Straße 2, 28359 Bremen, Germany; annika.gerken@mevis.fraunhofer.de (A.G.); alessa.hering@mevis.fraunhofer.de (A.H.); jan.moltz@mevis.fraunhofer.de (J.H.M.); 3 Diagnostic Image Analysis Group, Radboudumc, Geert Grooteplein Zuid 10, 6525 GA Nijmegen, The Netherlands; 4 Cluster of Excellence iFIT (EXC 2180) “Image-Guided and Functionally Instructed Tumor Therapies”, Faculty of Medicine, Eberhard Karls University, 72076 Tuebingen, Germany; 5 Max Planck Institute for Intelligent Systems, Max-Planck-Ring 4, 72076 Tuebingen, Germany; 6 Center of Dermato-Oncology, Department of Dermatology, Eberhard Karls University, Tuebingen University Hospital, Liebermeisterstraße 25, 72076 Tuebingen, Germany; thomas.eigentler@charite.de (T.K.E.); teresa.amaral@med.uni-tuebingen.de (T.A.); 7 Department of Dermatology, Venereology and Allergology, Charité—Universitätsmedizin Berlin, Freie Universität Berlin and Humboldt-Universität zu Berlin, Luisenstraße 2, 10117 Berlin, Germany; 8 Institute of Neuroradiology, Johannes Gutenberg University Hospital Mainz, Langenbeckstraße 1, 55131 Mainz, Germany; ahmed.othman@unimedizin-mainz.de

## Text Correction

There was an error in the original publication [1]. Section 2.4 in the manuscript and S2.2 in the supplementary should contain the information “and presence of new metastases in first follow-up CT” for the description of the clinical features used as input.

A correction has been made to Section 2.4 in the manuscript and S2.2 in the supplementary. The authors apologize for any inconvenience caused and state that the scientific conclusions are unaffected. This correction was approved by the Academic Editor. The original publication has also been updated.
