# Supplementary material for: Can Delta Radiomics Improve the Prediction of Best Overall Response, Progression-Free Survival, and Overall Survival of Melanoma Patients Treated with Immune Checkpoint Inhibitors?
Source: Cancers (Basel). 2024 Jul 26;16(15):2669. doi: 10.3390/cancers16152669 (PMC11312160; doi:10.3390/cancers16152669)
Supplement: Supplementary file 1 [file cancers-16-02669-s001.zip › cancers-3085522-supplementary.pdf]

Supplementary

S1. Scan parameters and CT vendor details.

Inhouse staging CTs were performed on four CT scanners (Sensation 64, SOMATOM Definition AS, SOMATOM Definition Flash, SOMATOM Force, Siemens Healthineers, Erlangen, Germany) and one PET-CT scanner (Biograph128, Siemens Healthineers, Erlangen, Germany). The inhouse whole-body staging protocol was used with a scan field from skull base to the middle of the femur with patients laid in a supine position, arms raised above the head. Scanning was performed during the portal-venous phase after administration of body-weight-adapted contrast medium through the cubital vein. Attenuation-based tube current modulation (CARE Dose, reference mAs 240) and tube voltage (120 kV) were applied. The following scan parameters were used: SOMATOM Definition AS: collimation 64 × 0.6 mm, rotation time 0.5 s, pitch 0.6; SOMATOM Definition Flash: collimation 128 × 0.6 mm, rotation time 0.5 s, pitch 1.0; SOMATOM Force: collimation 128 × 0.6 mm, rotation time 0.5 s, pitch 0.6; Sensation 64: collimation 64 × 0.6 mm, rotation time 0.5 s, pitch 0.6; Biograph128: collimation 128 × 0.6 mm, rotation time 0.5 s, pitch 0.8. Slice thickness as well as increment were set to 3 mm. A medium smooth kernel was used for image reconstruction. In total 22 external CTs were also included to account for a more realistic sample and reduce sample bias. Detailed information for contrast medium phase, tube current and tube voltage are not available for these cases. For a detailed distribution of CT vendors see Table S1.

Table S1: CT scanners and vendors.

			number of patients		
	scanner	vendor	baseline	follow-up	total
cohort					
inhouse	SOMATOM Definiton AS+	Siemens	31	27	58
	SOMATOM Definiton Flash	Siemens	2	4	6
	SOMATOM Force	Siemens	53	64	117
	Sensation 64	Siemens	21	20	41
	Biograph128	Siemens	24	24	48
external	Aquillion One	Canon	1		1
	Astelion	Canon	1	1	2
	Optima CT540	GE	2		2
	Brilliance 40	Philips		2	2

	Biograph64	Siemens	1		1
	Emotion 16	Siemens	1	2	3
	Scope	Siemens	1	1	2
	Sensation 64	Siemens	1		1
	SOMATOM Definition AS	Siemens	4		4
	SOMATOM Definition Edge	Siemens	2		2
	SOMATOM Definition Flash	Siemens	1		1
	SOMATOM Force	Siemens		1	1
total			146	146	292

S2. Detailed description of the radiomic feature extraction and aggregation, the machine learning model and model evaluation.

S2.1. Radiomic feature extraction and aggregation.

For each segmented lesion, 14 radiomic shape features, 18 first-order statistics features, and 1284 texture features were extracted using the reference standard for radiomics analysis, the Pyradiomics Python package, version 3.0.1. The non-shape features were extracted on three different image types: original image (93 features), image filtered with Laplacian of Gaussian (LoG) with σ=1,2,3,4,5 mm (465 features) and wavelet-transformed image (744 features). In total, 1316 features were extracted per lesion. As lesions can disappear or be newly formed at first follow-up, the delta features cannot be calculated on lesion-level, because most features do not have meaningful values for empty lesions. Therefore, we first aggregated all lesion features per patient and time point and then computed delta features on patient-level. Aggregation was performed by computing the sum of the feature values for shape features and the volume-weighted mean for all other features. We aggregated lesions in two ways: all lesions and only the up to ten largest lesions at baseline. In the latter case, at follow-up the same lesions were used as at baseline, unless they had disappeared. In addition to the delta features, a binary indicator of the presence of new lesions at follow-up was provided to the model. Automatic feature selection was applied during training to select those features that have a high correlation with the outcome on training data and little correlation with other selected features, using the Fast Correlation Based Filter for Feature Selection (FCBF) method.

S2.2. Machine learning model.

The machine learning (ML) models were built for seven different clinical endpoints: best overall response to therapy according to Response Evaluation Criteria In Solid Tumors (RECIST) 1.1 criteria (binarized: complete or partial response = response; stable or progressive disease = no response), progression-free survival after six, nine and twelve months and overall survival after six, nine and twelve months. For each endpoint, the total patient cohort was reduced to those patients for which the endpoint information was available. The excluded patients were censored. Three ML models were trained per endpoint: the first model was trained on clinical data only, using the following clinical features as input: age, gender, type of immunotherapy, localization of primary tumor, histological subtype of primary tumor, BRAF (v-Raf murine sarcoma viral oncogene homolog B1) V600E mutation status, baseline lactate dehydrogenase level, follow up lactate dehydrogenase level, baseline S100 level, follow up S100 level, number of metastatic organs in baseline CT, presence of cerebral metastases or hepatic metastases and presence of new metastases in first follow-up CT. The second model was trained on all clinical features and a subset of the aggregated radiomic features from the total tumor burden, that was automatically selected per fold. The third model was trained on all clinical features and a subset of the aggregated radiomic features from the ten largest metastases.

All ML models were trained in 10x5-fold cross validation (CV) with random assignment of patients to the folds to estimate the prediction performance. Per fold, the ML model pipeline consisted of four steps, of which steps 1-3 were performed based on the respective training set only: 1. **Pre-processing:** Ordinal encoding of nominal clinical features, imputation of missing clinical feature values (0.5 for binary features, median for all other features), standard normalization (zero mean, unit variance) of all features. 2. **Feature selection** using FCBF: Applied only to radiomic features, clinical features were always used. 3. **Training**: Fit of an extremely randomized forest. 4. **Validation**: Prediction of outcome on the current validation set and comparison to true outcome using AUC.

Repeated CV was chosen over simple CV to reduce the impact of the random assignment to folds (or data split) in the evaluation and therefore to obtain a more reliable estimate of the overall model

performance. A random forest (RF) was chosen as the core ML model because of its advantages such as the low need for hyper-parameter tuning and robustness to noisy variables. The extremely randomized forest variant was chosen, which alleviates the bias in split selection by randomly binarizing all variables before applying the splitting rules. We used the implementation provided in the scikit-learn Phyton package (version 0.24.2, ExtraTreesClassifier) with default parameters but enabled bootstrapping for building the trees.

S2.3. Model evaluation.

The area under the curve (AUC) of the receiver operating characteristic (ROC) curve was chosen as classification performance metric. We used bootstrapping with 1000 samples to estimate a 95% confidence interval (CI) for the mean AUC of the 10x5-fold CV of each model. We computed the mean AUC by pooling the predictions on all 5 folds and repeated this procedure for each of the 10 CV repetitions, using the same bootstrap sample on patient level per repetition. Per bootstrap sample, we then calculated the mean AUC across the 10 repetitions. We computed a mean ROC curve analogously with 95% CI by estimating the true positive rate of predictions via bootstrapping. Statistically significant superior performance of the extended model is achieved if the CI of the mean AUC of baseline and extended models do not overlap. Significant predictive capacity of a model following the outcome distribution is achieved if the lower bound of the CI is higher than 0.5.

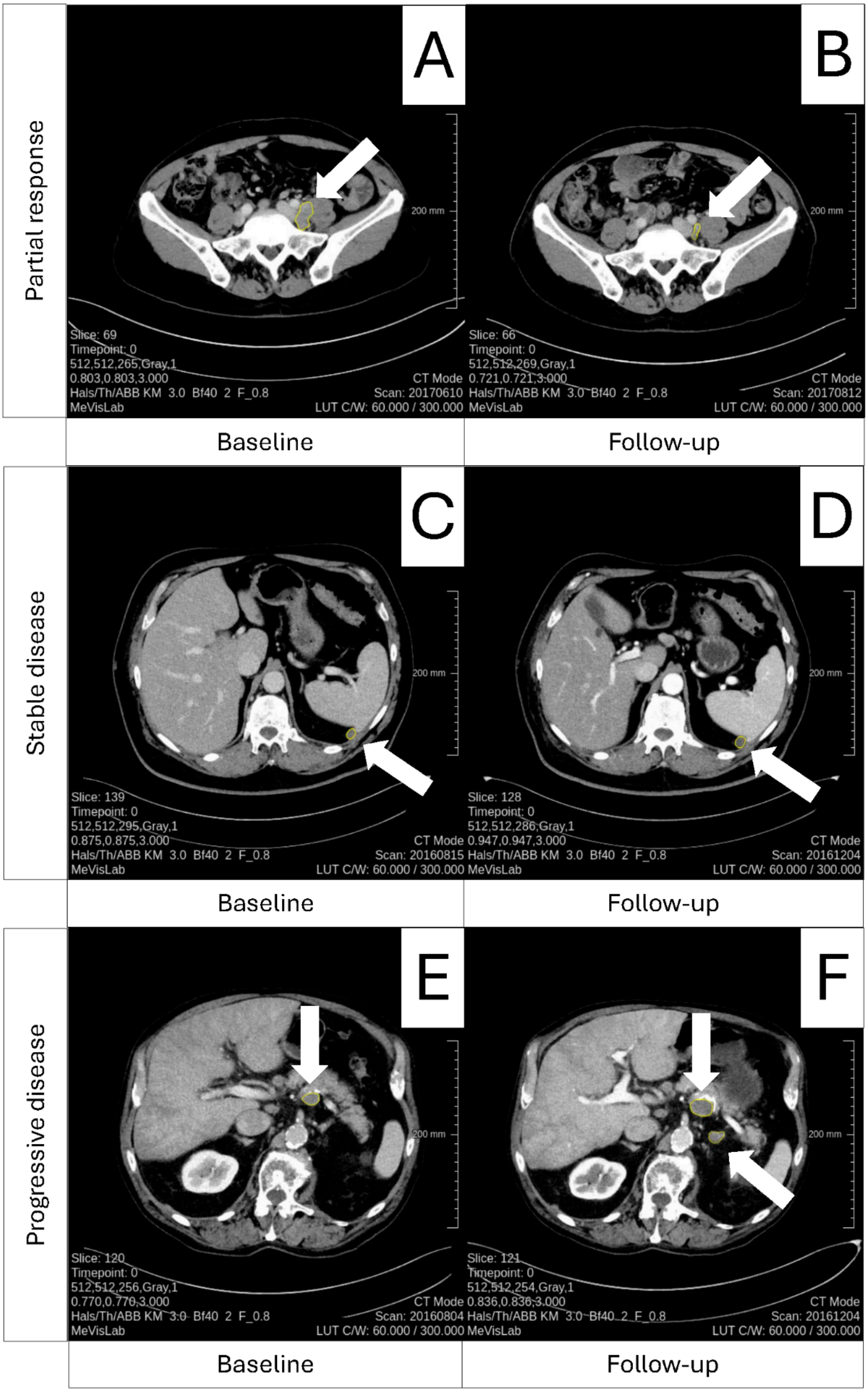

Figure S1: Examples of different timepoint responses: A, C, E show baseline CT imaging of three different patients. B, D, F show CT imaging of the first follow-up with timepoint responses "partial response", "stable disease" and "progressive disease", respectively.
